# Angulation and curvature of aortic landing zone affect implantation depth in transcatheter aortic valve implantation

**DOI:** 10.1038/s41598-024-61084-5

**Published:** 2024-05-06

**Authors:** Riccardo Gorla, Omar A. Oliva, Luca Arzuffi, Valentina Milani, Simone Saitta, Mattia Squillace, Enrico Poletti, Maurizio Tusa, Emiliano Votta, Nedy Brambilla, Luca Testa, Francesco Bedogni, Francesco Sturla

**Affiliations:** 1 Department of Clinical and Interventional Cardiology, IRCCS Policlinico San Donato, P.Zza Edmondo Malan 2, 20097 San Donato Milanese, Milan, Italy; 2 Scientific Directorate, IRCCS Policlinico San Donato, San Donato Milanese, Italy; 3 Department of Electronics, Information and Bioengineering, Politecnico di Milano, Milano, Italy; 4 3D and Computer Simulation Laboratory, IRCCS Policlinico San Donato, San Donato Milanese, Italy

**Keywords:** Aortic stenosis, TAVI, Innovation, Risk stratification, Interventional cardiology, Cardiac device therapy, Risk factors, Valvular disease, Predictive markers

## Abstract

In transcatheter aortic valve implantation (TAVI), final device position may be affected by device interaction with the whole aortic landing zone (LZ) extending to ascending aorta. We investigated the impact of aortic LZ curvature and angulation on TAVI implantation depth, comparing short-frame balloon-expanding (BE) and long-frame self-expanding (SE) devices. Patients (n = 202) treated with BE or SE devices were matched based on one-to-one propensity score. Primary endpoint was the mismatch between the intended (H_Pre_) and the final (H_Post_) implantation depth. LZ curvature and angulation were calculated based on the aortic centerline trajectory available from pre-TAVI computed tomography. Total LZ curvature (${k}_{LZ,tot}$) and LZ angulation distal to aortic annulus (${\alpha }_{LZ,Distal}$) were greater in the SE compared to the BE group (*P* < 0.001 for both). In the BE group, H_Post_ was significantly higher than H_Pre_ at both cusps (*P* < 0.001). In the SE group, H_Post_ was significantly deeper than H_Pre_ only at the left coronary cusp (*P* = 0.013). At multivariate analysis, ${\alpha }_{LZ,Distal}$ was the only independent predictor (OR = 1.11, *P* = 0.002) of deeper final implantation depth with a cut-off value of 17.8°. Aortic LZ curvature and angulation significantly affected final TAVI implantation depth, especially in high stent-frame SE devices reporting, upon complete release, deeper implantation depth with respect to the intended one.

## Introduction

Transcatheter aortic valve implantation (TAVI) has emerged as the treatment of choice for patients with severe aortic stenosis at high or intermediate surgical risk^1,2^. An increased aortic angulation, as evident in the so-called horizontal aorta, has already been included among the anatomical factors increasing the complexity of TAVI procedures^3^ and, in some reports, also affecting procedural success^4–6^. Based on computed tomography (CT), aortic angulation (AA) is generally defined on a coronal projection at the level of the aortic annulus as the angle between the horizontal (i.e., axial) plane and the plane of the aortic annulus^6,7^. Abramovitz et al.^6^ reported that an increased AA adversely influences acute procedural success of TAVIs performed by implanting self-expandable (SE) transcatheter heart valves (THVs) but not balloon-expandable (BE) THVs. Conversely, in a recent study, Medranda et al. suggested that AA does not affect TAVI outcomes using the new-generation BE (SAPIEN 3) or SE (CoreValve Evolut PRO) THVs^7^.

Based on this conflicting evidence, we hypothesized that the “whole” aortic landing zone, which may extend up to the proximal tract of the ascending aorta in case of TAVI with SE THVs, may be relevant in addition to other anatomical features (e.g., bicuspid aortic valve, elliptic aortic annulus and ascending aorta diameter) in predicting final THV position and outcome.

To test this hypothesis, we retrospectively quantified the anatomy of the aortic landing zone, namely in terms of curvature and angulation, in patients receiving SE and BE THVs and evaluated the impact of these features on the final THV implantation depth in the two groups.

## Materials and methods

### Study design and data collection

This is an observational, retrospective and single-center registry enrolling patients with severe aortic stenosis treated from December 2016 to September 2021 with one of the following THVs: Evolut R/Pro (Medtronic, Minneapolis, MN, USA), Portico (Abbott, Minneapolis, MN, USA), Myval (Meril Life Sciences Pvt Ltd, Vapi, Gujarat, India), and Sapien 3 (Edwards Lifesciences, Irvine, CA, USA).

Valve-in-valve TAVI, and bicuspid aortic valve were exclusion criteria. Patients were divided in two groups: (i) patients implanted with a BE short stent frame THV (Myval and Sapien 3) and (ii) patients receiving a SE long stent frame THV (Evolut R/Pro and Portico).

All the collected records on the enrolled patients were retrieved from the institutional TAVI database, including data regarding baseline conditions, preprocedural CT angiography, echocardiography, TAVI procedure and outcome. For each patient, consensus to proceed with TAVI was reached following Heart Team discussion, as per protocol of our Institute.

One-to-one propensity score matching was employed to balance the comparison between the two groups and remove the potential bias due to baseline characteristics, namely age, Society of Thoracic Surgeons (STS) score, body surface area (BSA), calcium volume 800 HU and aortic angulation. The propensity score was created using a multivariable logistic regression model. Matching was performed with a fixed ratio 1:1 optimal matching protocol without replacement and using a caliper width equal to 0.28 of the logit of the standard deviation.

The study was conducted in accordance with the Declaration of Helsinki and approved by the local Ethics Committee of IRCCS Ospedale San Raffaele (protocol code “AI4TAVI”, No. 33/INT/2023, accepted on March 15th, 2023); informed consent was waived because of the retrospective nature of the study and the anonymized data analysis.

### Study endpoint and definitions

The primary endpoint of the study was the mismatch (∆H) between the intended (H_Pre_) and the final (H_Post_) implantation depth of each TAVI THV, defined for each dataset as:

$$\Delta H={H}_{Post}-{H}_{Pre}$$

Implantation depth was defined as the maximal distance between the intraventricular edge of the bioprosthesis and the aortic annulus at the level of both the non-coronary cusp (NCC) and left coronary cusp (LCC)^8^ calculated from the implantation projection where the inflow edges are aligned. $\Delta H$ was computed at both cusps, yielding ${\Delta H}_{NCC}$ and ${\Delta H}_{LCC}$, respectively, and their mean value ${\Delta H}_{mean}$. The choice of implantation projection was left to the operator discretion; for SE-devices, both three-cusps view and cusp overlap view were employed, with the latter increasingly used after 2019; for BE-devices, three cusps view was employed in all cases.

For BE valves, the intended implantation depth was measured with the valve fully closed, before deployment under rapid pacing. For SE valves, the intended implantation depth was measured with the valve opened up to the non-recapture point, prior to complete release.

Device success after TAVI was defined according to VARC-3 definition upon fulfilling all the following criteria^9^: technical success, freedom from mortality, freedom from surgery or intervention related to the THV device or to a major vascular or access-related or cardiac structural complication, intended performance of the THV (mean gradient < 20 mmHg, peak velocity < 3 m/s, Doppler velocity index ≥ 0.25, and less than moderate aortic regurgitation). All these endpoints were evaluated during the index hospitalization.

### CT acquisitions and image processing

CT angiography was acquired on a 64-row multidetector scanner (SOMATOM Definition, Siemens Healthineers, Erlangen, Germany). Image sequential acquisition was performed with retrospective ECG-gating. The optimal systolic reconstruction (BestSyst) was considered for the subsequent analysis. Due to the retrospective nature of the study, patients with CT imaging not available were excluded from the analysis. Pixel spacing ranged from 0.26 × 0.26 mm^2^ to 0.87 × 0.87 mm^2^, while slice thickness ranged between 0.25 and 1 mm.

**Figure 1:**
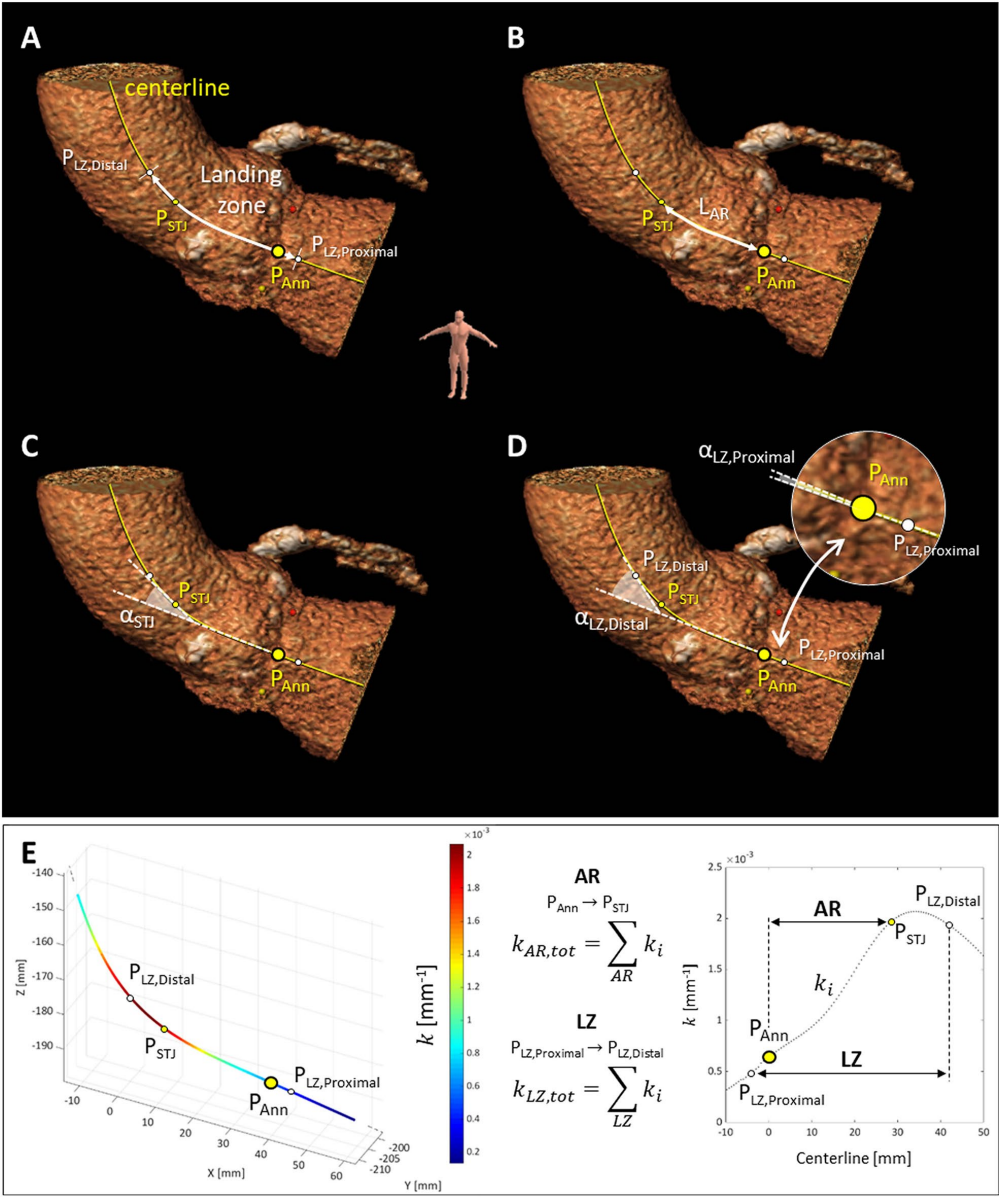
Landing zone analysis. Identification of the landing zone (LZ) on the aortic centerline for a SE valve (**A**), definition of the AR length (L_AR_, **B**) and extraction of STJ plane angulation (α_STJ_, **C**), proximal LZ plane angulation (α_LZ,Proximal_) and distal LZ plane angulation (α_LZ,Distal_) with respect to the aortic annulus plane (**D**). Quantification of the cumulative absolute curvature of the aortic centerline (**E**) in the AR (${k}_{AR,tot}$) and LZ (${k}_{LZ,tot}$) regions, respectively. Ann, aortic annulus; AR, aortic root; LZ, landing zone; STJ, sinotubular junction.

Each dataset was imported in 3mensio Structural Heart (version 8.2, Pie Medical Imaging BV, Maastricht, The Netherlands) and post-processed by a qualified operator. Aortic centerline was automatically detected and verified by the user through multiplanar reconstruction views, with the possibility to adjust the position of the control points lying on the centerline (Fig. 1.A). The annulus plane, which is the reference for all the measurements along the centerline, was defined as the plane passing through the user-specified position of the three nadirs of the aortic leaflets (P_Ann_ on the centerline). The sinotubular junction (STJ) was identified annotating its position along the centerline with respect to the annular plane (P_STJ_). Several measurements (e.g., area, perimeter, and diameters) were extracted as part of the standard preprocedural evaluation of the aortic root (AR) anatomy^10^.

Aortic angulation was calculated on CT angiography from the implantation projection in which the three coronary cusps were aligned and was defined as the angle between the horizontal plane and the plane of the aortic annulus^5^.

Finally, the profile of the centerline was exported from 3 mensio as a set of points, whose 3D coordinates were stored in a file with extensible markup language (xml) format.

### Analysis of the aortic landing zone

The aortic landing zone was assessed in terms of the geometric features of its centerline, which were analyzed using a dedicated script written in Matlab (The MathWorks Inc., Natick, MA, USA)^11^. To this purpose, each centerline profile was described in the parametric form ${\varvec{r}}\left(s\right)=\left(x\left(s\right),y\left(s\right),z\left(s\right)\right)$ as a function of its arc length ($s$) and interpolated through non-uniform rational basis splines (NURBS). For each dataset, the nominal LZ for TAVI was defined in accordance with the nominal height of each THV, which varies according to the label size. Specifically, within the SE long stent frame group it ranges between 50 and 53 mm for Portico, and between 45 and 46 mm for Evolut R/Pro. Conversely, in the BE short stent frame group, the nominal height ranges between 17 and 21 mm for Myval and between 15.5 and 22.5 mm for Sapien 3. Further details are available in Supplementary Table S1. The initial proximal point of the nominal LZ, i.e., P_LZ,Proximal_ (Fig. 1.A) was automatically positioned 4 mm below the annular point (P_Ann_) for all the analyzed centerlines, as representative of target implantation depth in TAVR (9,10). The distal LZ extremity, i.e., P_LZ,Distal_, was defined on the centerline taking the THV-specific nominal height into account.

AR length (L_AR_) was measured as the length of the portion of the centerline profile between the annulus plane and the STJ plane (Fig. 1.B).

The following angular characteristics were extracted from the centerline trajectory (Fig. 1): the STJ angulation ${\alpha }_{STJ}$ with respect to the aortic annulus, expressed as the angle between the tangent unit vector to the centerline on the annulus plane and the corresponding tangent unit vector on the STJ plane (Fig. 1.C); the angulation of both the distal and proximal LZ extremities with respect to the aortic annulus, namely ${\alpha }_{LZ,Distal}$ and ${\alpha }_{LZ,Proximal}$ respectively, each one defined as the angle between the tangent unit vector to the centerline on the distal/proximal LZ plane and the corresponding tangent unit vector on the annulus plane (Fig. 1.D).

Also, on each point of the centerline, the tangent ($\overrightarrow{T}(s)$), normal ($\overrightarrow{N}(s)$), and binormal ($\overrightarrow{B}(s)$) unit vectors were calculated according to the Frenet–Serret frame^12^ (Supplementary Material) to compute the pointwise absolute value of curvature $k$, which quantifies the local bending of the centerline, i.e., the deviation of the curve from a straight line, expressed in mm^−1^ (Fig. 1.E). Accordingly, the cumulative $k$ was calculated along the AR and LZ regions, namely as ${k}_{AR,tot}$ and ${k}_{LZ,tot}$, respectively.

### Statistical analysis

Normal distribution was checked by Kolgomorov-Smirgov and Shapiro–Wilk tests. Continuous variables following a normal distribution are reported as mean and standard deviation; otherwise, median and interquartile range are presented. Covariates following a normal distribution were compared using unpaired Student’s t-test, while Mann–Whitney U tests were used to compare continuous variables with skewed distribution. Categorical and dichotomous variables are presented as counts and percentages and were compared by Pearson chi-square or Fisher exact tests, as appropriate.

Receiver-operating characteristic (ROC) curve analysis was employed to investigate the predictive value of the analyzed LZ features on the mismatch ${\Delta H}_{mean}$ in implantation depth. To this purpose, the study population was dichotomized in terms of ${\Delta H}_{mean}$ classifying each dataset as below or above the pre-defined threshold of ${\overline{\Delta H} }_{mean}$, i.e., the average value of ${\Delta H}_{mean}$ over the study population. For each feature, the area under the curve (AUC) was calculated and the best cutoff was evaluated using the maximal Youden Index.

Univariate and multivariate logistic regression analysis were carried out to sort out the analyzed variables according to their potential impact on the binary endpoint of TAVI implantation depth. Variables with *P* values < 0.05 at univariate analysis were entered simultaneously in the multivariate logistic regression analysis.

All P-values were two-sided with values < 0.05 considered statistically significant. Analyses were performed using SPSS 28.0 statistical analysis software (IBM Italia, Milano, Italy).

## Results

Of the 784 TAVI patients eligible for the study, 143 received a BE THV while 641 received a SE THV (Figure S1). Based on the propensity score matching, 266 patients were selected and matched obtaining 133 patients for BE and SE group, respectively. The results of propensity score matching are detailed in Table S2 while baseline main characteristics before (unmatched) and after (matched) propensity score matching are summarized in Table S3.

Due to unavailability of CT imaging for 34 patients, the final matched study cohort included 202 TAVI patients, i.e., 101 for each group; baseline characteristics are summarized in Table 1. Differences between the two groups were not statistically significant in terms of age, cardiovascular risk factors, STS score, creatinine clearance, calcium score, index of eccentricity and aortic angulation. Patients in the BE group showed lower left ventricle ejection fraction (*P* < 0.001), lower mean AV gradient (*P* = 0.002) and, on CT angiography, a larger and higher aortic root as compared to the patients in the SE group. Also, in the BE group both α_STJ_ and k_AR,tot_ were higher (*P* = 0.024 and *P* = 0.035, respectively) than in the SE group.

**Table 1:** Baseline patient characteristics.

Variables	Overall (n = 202)	BE (n = 101)	SE (n = 101)	*P* value^§^
Age (years)	81 (77, 85)	81 (78, 86)	81 (77, 85)	0.56
Female sex	64 (31.7)	26 (25.7)	38 (37.6)	0.07
BSA (m^2^)	1.86 ± 0.19	1.85 ± 0.19	1.86 ± 0.20	0.74
Hypertension	151 (74.8)	78 (77.2)	73 (72.3)	0.42
Diabetes	57 (28.2)	30 (29.7)	27 (26.7)	0.64
Dyslipidemia	81 (40.1)	38 (37.6)	43 (42.6)	0.47
COPD	27 (13.4)	12 (11.9)	15 (14.9)	0.54
CAD	48 (23.8)	20 (19.8)	28 (27.7)	0.19
Prior CABG	23 (11.4)	8 (7.9)	15 (14.9)	0.12
Prior AMI	15 (7.4)	9 (8.9)	6 (5.9)	0.42
Prior AF	58 (28.7)	31 (30.7)	27 (26.7)	0.53
STS score (%)	3.2 (2.1, 5.6)	3.2 (2.4, 5.8)	3.2 (2.0, 5.3)	0.38
Creatinine clearance (mL/min/1.73 m^2^)	58.5 (43.0, 74.0)	60.5 (43.8, 72.3)	57.0 (43.0, 73.3)	0.35
Haemoglobin (g/dL)	12.5 ± 1.9	12.6 ± 2.0	12.4 ± 1.8	0.62
Ejection fraction (%)	55.0 (44.0, 63.0)	51.0 (40.0, 60.0)	58.5 (50.0, 65.0)	** < 0.001**
Mean AV gradient (mmHg)	42.6 ± 15.0	38.9 ± 14.4	45.7 ± 14.8	**0.002**
Aortic regurgitation ≥ moderate	35 (17.3)	18 (17.8)	17 (16.8)	0.85
LM height (mm)	15.8 ± 3.8	16.1 ± 3.8	15.5 ± 3.8	0.21
RCA height (mm)	19.5 ± 3.6	19.7 ± 3.4	19.2 ± 3.9	0.27
Annulus minimal diameter (mm)	22.5 ± 3.0	23.4 ± 2.7	21.5 ± 2.9	** < 0.001**
Annulus maximal diameter (mm)	28.4 ± 2.9	29.3 ± 2.8	27.4 ± 2.6	** < 0.001**
Annulus mean diameter (mm)	25.4 ± 2.6	26.3 ± 2.5	24.5 ± 2.4	** < 0.001**
Annulus perimeter (mm)	80.0 ± 8.2	82.7 ± 8.1	77.2 ± 7.4	** < 0.001**
Annulus area (mm^2^)	496.5 ± 103.3	531.2 ± 101.7	461.7 ± 93.1	** < 0.001**
LVOT diameter (mm)	24.9 ± 3.2	26.0 ± 3.1	23.9 ± 3.1	** < 0.001**
Valsalva diameter (mm)	34.0 ± 3.8	34.7 ± 3.8	33.4 ± 3.7	**0.004**
Calcium volume 800 HU (mm^3^)	249 (119, 474)	268 (126, 499)	184 (112, 470)	0.39
Aortic angulation (°)	48.1 ± 9.5	48.4 ± 9.8	47.8 ± 9.3	0.63
Index of eccentricity	0.20 (0.17, 0.25)	0.20 (0.16, 0.24)	0.21 (0.17, 0.26)	0.17
L_AR_ (mm)	22.4 ± 3.6	23.1 ± 3.5	21.7 ± 3.6	**0.009**
k_AR,tot_ (10^–1^·mm^-1^)	0.37 (0.26, 0.51)	0.41 (0.27, 0.53)	0.35 (0.25, 0.45)	**0.035**
k_LZ,tot_ (10^–1^·mm^-1^)	0.83 (0.41, 1.27)	0.41 (0.26, 0.56)	1.23 (1.00, 1.48)	** < 0.001**
α_STJ_ (°)	9.1 (5.7, 13.0)	10.7 (6.2, 14.2)	8.3 (5.1, 12.1)	**0.024**
α_LZ,Proximal_ (°)	2.6 (1.4, 4.3)	2.9 (1.7, 4.8)	2.5 (1.3, 3.9)	0.16
α_LZ,Distal_ (°)	15.8 (6.9, 28.6)	7.4 (4.7, 11.0)	28.5 (21.5, 37.1)	** < 0.001**

Values are mean ± SD, median (IQR) or n (% of column total).

*AF*, atrial fibrillation; *AMI*, acute myocardial infarction; *AR*, aortic root; *AV*, aortic valve; *BE*, balloon-expandable; *BSA*, body surface area; *CABG*, coronary artery bypass grafting; *CAD*, coronary artery disease; *COPD*, chronic obstructive pulmonary disease; *HU*, Hounsfield Units; *k*_*AR,tot*_, total (cumulative) curvature of the aortic root centerline; *k*_*LZ,tot*_, total (cumulative) curvature of the landing zone centerline; *LZ*, landing zone; *L*_*AR*_, aortic root length; *LM*, left main; *LVOT*, left ventricular outflow tract; *RCA*, right coronary artery; *SE*, self-expandable; *STJ*, sinotubular junction; *STS*, Society of Thoracic Surgeons; *α*_*STJ*_, angulation of the STJ plane with respect to the aortic annulus plane; *α*_*LZ,Proximal*_, angulation of the proximal LZ plane with respect to the aortic annulus plane; *α*_*LZ,Distal*_, angulation of the distal LZ plane with respect to the aortic annulus plane.

^§^BE vs. SE.

Significant values (*P*<0.05) are in bold.

Focusing on the THV-specific landing zone, in the SE group mean k_LZ,tot_ was three times greater than in the BE group (1.23 10^–1^·mm^−1^ vs. 0.41 10^–1^·mm^-1^, *P* < 0.001), mean α_LZ,Distal_ was almost four times wider (28.5° vs. 7.4°, *P* < 0.001), but α_LZ,Proximal_ remained comparable (*P* = 0.16). Baseline characteristics clustered according to ${\Delta H}_{mean}$ are available in Table S4.

The THVs employed for TAVI are detailed in Table 2; transfemoral access was used in the majority of patients (88.6%) while subclavian access route was more frequent in the SE group (*P* = 0.03), which also reported larger contrast volume (*P* = 0.02), longer radiation time (*P* < 0.001) and higher rates of predilatation (*P* = 0.001) and postdilatation (*P* < 0.001) with respect to the BE group. Negligible differences were noted between BE and SE in terms of vascular complications, stenting of the access site and concomitant percutaneous coronary intervention (PCI).

**Table 2:** Procedural data and clinical in-hospital outcome.

Variables	Overall (n = 202)	BE (n = 101)	SE (n = 101)	*P* value^§^
Implanted THV type				
Myval	81 (40.1)	81 (80.2)	–	–
Sapien 3	20 (9.9)	20 (19.8)	–	–
Evolut Pro	22 (10.9)	–	22 (21.8)	–
Evolut R	53 (26.2)	–	53 (52.5)	–
Portico	26 (12.9)	–	26 (27.7)	–
Femoral route	179 (88.6)	91 (90.1)	88 (87.1)	0.51
Subclavian route	11 (5.4)	2 (2.0)	9 (8.9)	**0.03**
EPS	5 (2.5)	3 (3.0)	2 (2.0)	0.67
Any vascular complications	11 (5.4)	7 (6.9)	4 (4.0)	0.35
PTA with stenting of access site	10 (5.0)	7 (6.9)	3 (3.0)	0.19
PCI with stenting	17 (8.4)	10 (9.9)	7 (6.9)	0.45
Predilatation	82 (40.6)	32 (31.7)	50 (49.5)	**0.001**
Implantation depth				
NCC H_Pre_ (mm)	7.0 (5.0, 9.0)	8.0 (7.0, 10.0)	5.0 (4.0, 8.0)	** < 0.001**
LCC H_Pre_ (mm)	8.0 (6.0, 10.0)	9.0 (8.0, 11.0)	7.0 (5.0, 9.0)	** < 0.001**
NCC H_Post_ (mm)	5.0 (4.0, 7.0)	5.0 (4.0, 6.0)	7.0 (4.6, 8.0)	** < 0.001**
LCC H_Post_ (mm)	6.0 (4.0, 8.0)	5.0 (4.0, 6.0)	8.0 (6.0, 10.0)	** < 0.001**
NCC ∆H (mm)	− 1.0 (− 4.0, 1.0)	− 4.0 (− 6.0, − 2.0)	0.0 (− 1.0, 2.5)	** < 0.001**
LCC ∆H (mm)	− 1.0 (− 5.0, 1.0)	− 5.0 (− 7.0, − 2.0)	1.0 (0.0, 3.0)	** < 0.001**
${\Delta H}_{mean}$(mm)	− 1.5 ± 3.7	–4.2 ± 2.5	1.1 ± 2.6	** < 0.001**
${\Delta H}_{mean}<{\overline{\Delta H} }_{mean}$	94 (46.5)	85 (84.2)	9 (8.9)	** < 0.001**
${\Delta H}_{mean}\ge {\overline{\Delta H} }_{mean}$	108 (53.5)	16 (15.8)	92 (91.1)	** < 0.001**
Postdilatation	58 (28.7)	6 (5.9)	52 (51.5)	** < 0.001**
Emergent cardiac surgery	0 (0.0)	0 (0.0)	0 (0.0)	–
Need for second valve	0 (0.0)	0 (0.0)	0 (0.0)	–
Contrast volume (mL)	150 (120, 180)	140 (111, 170)	150 (125, 184)	**0.02**
Radiation time (min)	19.4 (14.6, 26.4)	18.1 (12.7, 23.5)	22.2 (16.1, 28.5)	** < 0.001**
In-hospital outcome				
Ejection fraction (%)	56.0 (50.0, 63.0)	55.0 (45.0, 61.0)	58.5 (52.3, 65.0)	**0.012**
Mean gradient (mmHg)	7.0 (5.0, 9.0)	7.0 (6.0, 9.0)	7.0 (5.0, 10.0)	0.19
PVL absent/trivial	89 (44.1)	52 (51.5)	37 (36.6)	**0.034**
PVL mild	89 (44.1)	38 (37.6)	51 (50.5)	0.09
PVL > moderate	17 (8.4)	5 (5.0)	12 (10.8)	0.13
Device success	187 (92.6)	91 (90.1)	96 (95.1)	0.28
PPI	31 (15.3)	15 (14.9)	16 (15.8)	0.85
Stroke*	4 (2.0)	1 (1.0)	3 (3.0)	0.62
In-hospital mortality	1 (0.5)	1 (1.0)	0 (0.0)	0.28

Values are mean ± SD, median (IQR) or n (% of column total). Mismatch in implantation depth (∆H) calculated as H_Post_—H_Pre_; * Including not disabling stroke.

*EPS*, embolic protection system; *H*_*Pre*_, final implantation depth; *H*_*Pre*_, pre-implantation intended depth; *LCC*, left coronary cusp; *NCC*, non-coronary cusp; *PCI*, percutaneous coronary intervention; *PPI*, permanent pacemaker implantation; *PTA*, percutaneous transluminal angioplasty; *PVL*, paravalvular leakage; *∆H*, variation of implantation depth.

^§^BE vs. SE.

Significant values (*P*<0.05) are in bold.

**Figure 2:**
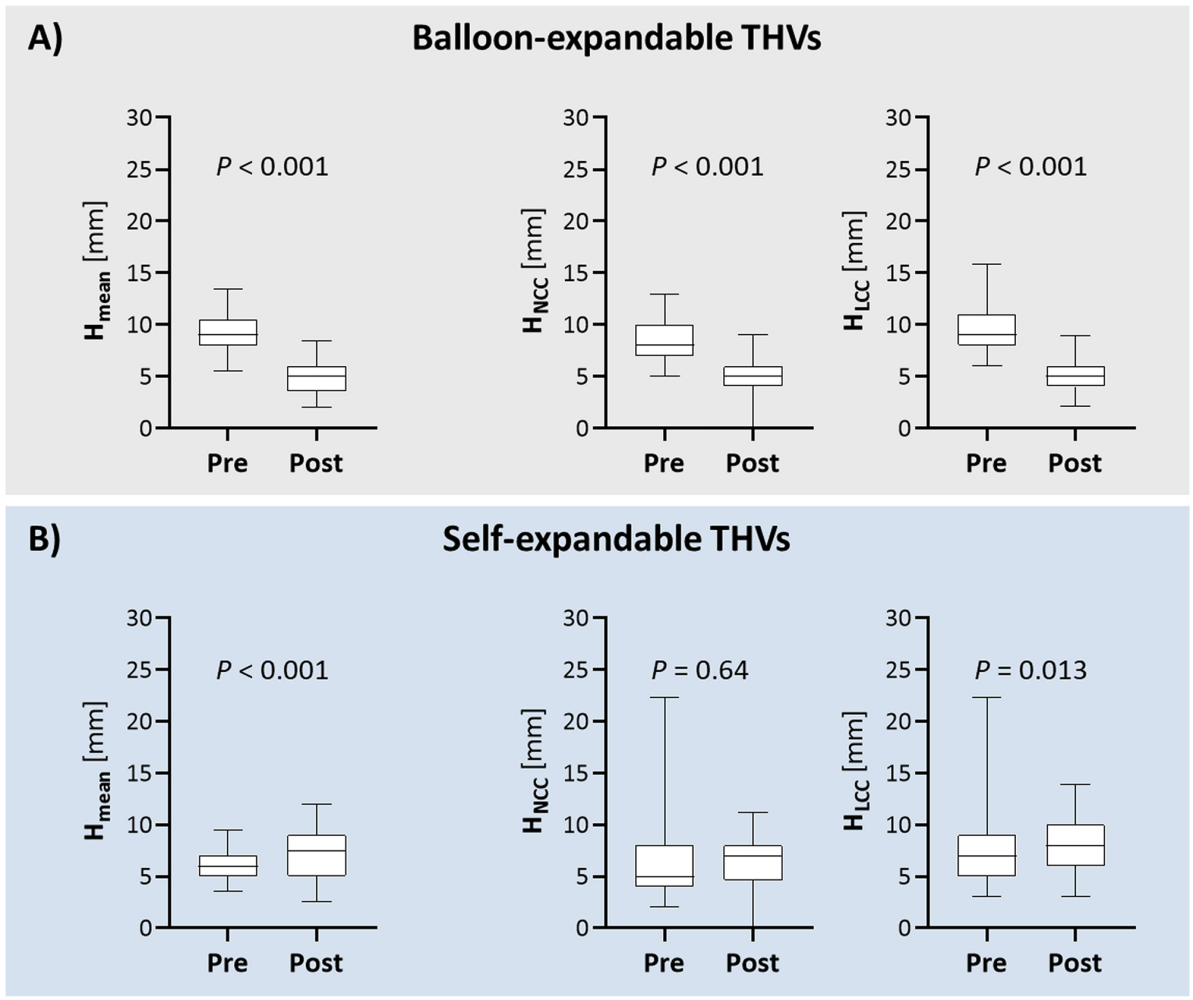
Box and whiskers plots of the intended (Pre) and final (Post) implantation depth (H) reported within BE (**A**) and SE (**B**) groups as mean (H_mean_), NCC (H_NCC_) and LCC (H_LCC_) values. BE, balloon-expandable; LCC, left coronary cusp; NCC, non-coronary cusp; SE, self-expandable; THV, transcatheter heart valve.

In terms of intended and final implantation depth (i.e., H_Pre_ and H_Post_, respectively), BE and SE THVs show a different behavior (Fig. 2). In the BE group, H_Post_ was significantly higher than H_Pre_ at both LCC and NCC cusps (*P* < 0.001), so that ${\Delta H}_{NCC}$ and ${\Delta H}_{LCC}$ were both negative. Instead, in the SE group, H_Post_ was significantly deeper than H_Pre_ at the LCC cusp (*P* = 0.013) but not at the NCC (*P* = 0.64) one.${\overline{\Delta H} }_{mean}$, i.e., the average of ${\Delta H}_{mean}$ over the entire study cohort, was equal to − 1.5 mm; when computed separately for each group, it was equal to -4.2 mm and 1.1 mm in the BE and SE group, respectively (*P* < 0.001).

**Figure 3:**
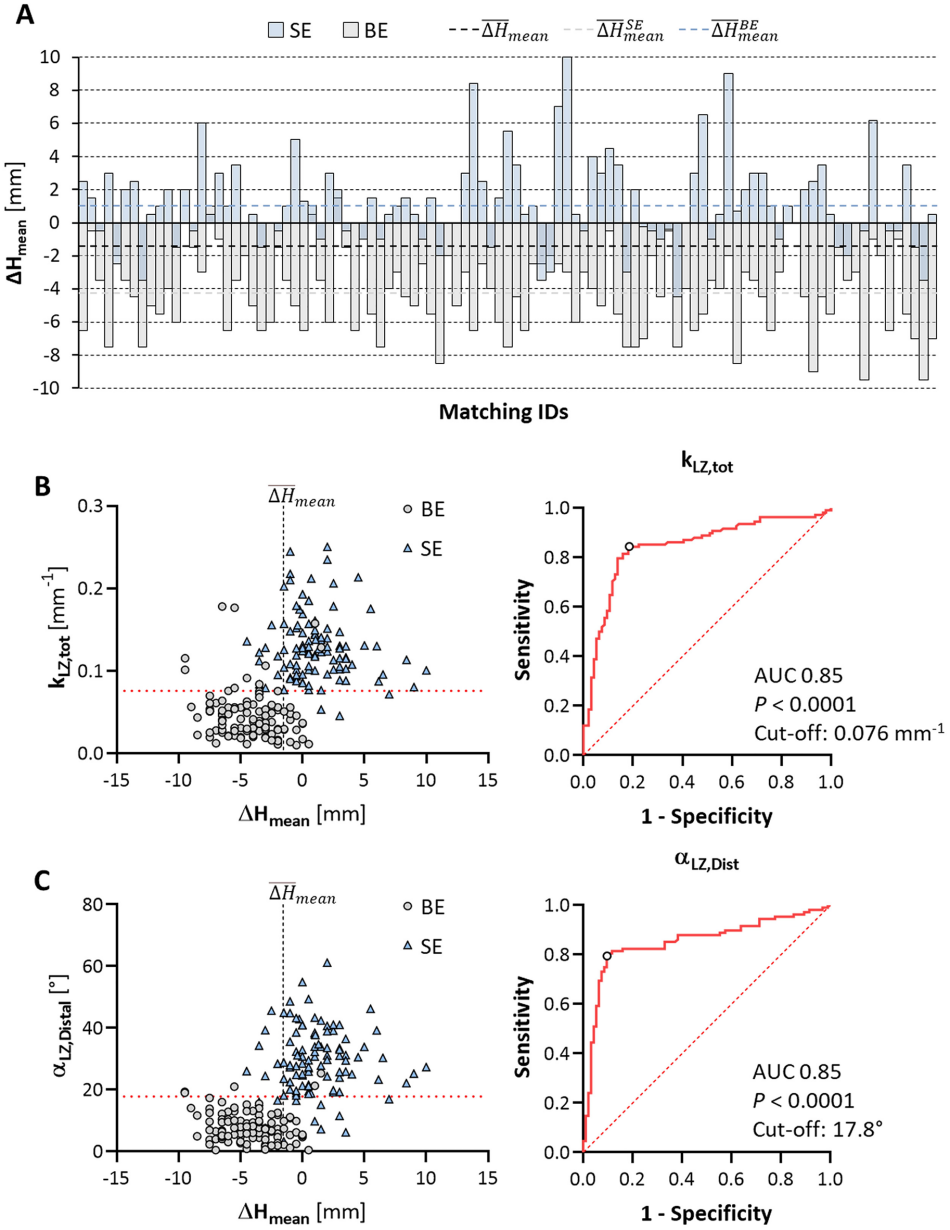
Bar plot of the patient-specific mismatch in the mean implantation depth ${\Delta H}_{mean}$ (**A**) within BE and SE groups, reporting data as paired values according to the results of one-to-one propensity score matching. Scatter plots of the association of (**B**) k_LZ,tot_ curvature and (**C**) α_LZ,Distal_ angulation with ${\Delta H}_{mean}$ and ROC curves evaluating the predictive value of each landing zone characteristic on the final variation of THV implantation depth. Other abbreviations as in Figs. 1 and 2.

Hence, on average, the final implantation depth was higher than the desired one (∆H_mean_ < 0) with BE THVs and deeper (∆H_mean_ > 0) with SE THVs (Fig. 3A). More in detail, we observed ${\Delta H}_{mean}<{\overline{\Delta H} }_{mean}$ in 84.2% (85/101) of BE THV recipients, but ${\Delta H}_{mean}\ge {\overline{\Delta H} }_{mean}$ in 91.1% (92/101) of SE THV recipients. Procedural data and clinical in-hospital outcome clustered according to ${\Delta H}_{mean}$ are reported in Table S5.

At ROC analysis (Table 2), both k_LZ,tot_ (Fig. 3B) and α_LZ,Distal_ (Fig. 3C) reported the highest predictive value (*P* < 0.001) on ${\Delta H}_{mean}$, reporting the same AUC equal to 0.85 and cut-off values of 0.76 10^–1^·mm^−1^ and 17.8°, respectively. Both variables were found to have the strongest association (*P* < 0.001) with ${\Delta H}_{mean}$ at univariate logistic regression analysis (Table 3). Correct and incorrect classifications are detailed in the Supplementary Table S6.

**Table 3:** Univariate and multivariate logistic regression analysis of the aortic root dimensions and landing zone features associated with implant height variation after TAVI.

Variables	ROC		Univariate (n = 202)		Multivariate (n = 202)	
	AUC	*P* value	OR (95% CI)	*P* value	OR (95% CI)	*P* value
Calcium Score 800 HU	0.57	0.11	0.99 (0.99 ÷ 1.00)	0.13		
Aortic angulation	0.50	0.99	1.00 (0.97 ÷ 1.03)	0.89		
Annulus minimal diameter	0.62	**0.003**	0.86 (0.77 ÷ 0.95)	**0.003**	1.29 (0.96 ÷ 1.72)	0.09
Annulus mean diameter	0.65	** < 0.001**	0.82 (0.73 ÷ 0.92)	**0.001**	0.66 (0.37 ÷ 1.19)	0.17
Annulus maximal diameter	0.65	** < 0.001**	0.84 (0.76 ÷ 0.93)	**0.001**	0.96 (0.69 ÷ 1.35)	0.82
Annulus perimeter	0.64	** < 0.001**	0.94 (0.91 ÷ 0.98)	**0.001**	1.23 (0.87 ÷ 1.73)	0.25
Annulus area	0.64	** < 0.001**	0.61 (0.45 ÷ 0.81)	**0.001**	0.61 (0.51 ÷ 7.43)	0.70
LVOT diameter	0.65	** < 0.001**	0.85 (0.77 ÷ 0.93)	**0.001**	0.77 (0.57 ÷ 1.03)	0.08
Valsalva diameter	0.59	**0.04**	0.94 (0.87 ÷ 1.02)	0.15		
L_AR_	0.60	**0.01**	0.89 (0.82 ÷ 0.96)	**0.004**	0.92 (0.81 ÷ 1.05)	0.21
k_AR,tot_	0.58	0.07	0.0 (0.0 ÷ 23.1)	0.14		
k_LZ,tot_	0.85	** < 0.001**	E^14^ (E^10^ ÷ E^17^)	** < 0.001**	E^+5^ (0.2 ÷ E^+11^)	0.15
α_STJ_	0.58	0.06	0.97 (0.92 ÷ 1.01)	0.16		
α_LZ,Proximal_	0.54	0.31	1.07 (0.97 ÷ 1.18)	0.21		
α_LZ,Distal_	0.85	** < 0.001**	1.15 (1.11 ÷ 1.20)	** < 0.001**	1.11 (1.04 ÷ 1.19)	**0.002**

*AR*, aortic root; *HU*, Hounsfield Units; *L*_*AR*_, aortic root length; *LVOT*, left ventricular outflow tract; *LZ*, landing zone; *k*_*AR,tot*_, total (cumulative) curvature of the aortic root centerline; *k*_*LZ,tot*_, total (cumulative) curvature of the landing zone centerline; *α*_*STJ*_, angulation of the STJ plane with respect to the aortic annulus plane; *α*_*LZ,Proximal*_, angulation of the proximal LZ plane with respect to the aortic annulus plane; *α*_*LZ,Distal*_, angulation of the distal LZ plane with respect to the aortic annulus plane; *STJ*, sinotubular junction.

Significant values (*P*<0.05) are in bold.

At multivariate logistic regression analysis, α_LZ,Distal_ arose as the only independent predictor (OR: 1.11; 95% CI 1.04 to 1.19, *P* = 0.002) of positive ${\Delta H}_{mean}$, i.e., of a final implantation depth deeper than the initially intended one.

Overall device success was satisfying and equal to 92.6% without significant differences between BE and SE groups (*P* = 0.28, Table 2), also in terms of permanent pacemaker implantation (PPI, *P* = 0.85), stroke (*P* = 0.62) and in-hospital mortality (*P* = 0.28). Ejection fraction remained higher in SE group (*P* = 0.012), as at baseline. Rate of absent/trivial PVL was higher in BE group (51.5 vs. 36.6%, *P* = 0.034), though the rate of PVL ≥ moderate remained comparable (5.0% in BE vs. 10.8% in SE, *P* = 0.13).

## Discussion

The main findings of the present study can be summarized as follows: (i) curvature and angulation of the aortic landing zone may significantly affect the final implantation depth of the THV in TAVI; (ii) high-frame SE valves, due to their nominal height, proved to be more sensitive than BE valves to the landing zone anatomy, with the interaction of the upper part of the device with the proximal ascending aorta potentially explaining SE deeper and asymmetrical implantation depth upon complete release.

The so-called horizontal aorta has been considered a debated factor in the recent years negatively affecting procedural success with SE valves according to some authors^3–6^, whereas no significant differences in outcomes between BE and SE devices were reported by others^7,13^.

The technical challenge of TAVI with SE devices (i.e., Portico and Evolut-R) in the horizontal aorta is represented by the difficulty to perform valve release maintaining the device coaxial to the aortic root (i.e., perpendicular to the aortic annulus) over the entire procedure. This may lead to an increased or asymmetrical implantation depth between NCC and LCC, though the issue is not systematically observed in all the horizontal anatomies as confirmed by the discrepancy of the data reported in the clinical literature. In this scenario, considering only the angulation of the aortic annulus may be too simplistic.

Therefore, to provide additional insight into the factors potentially affecting implantation depth in TAVI, we deepened the characterization of the aortic landing zone in terms of both curvature and angulation. Also, to balance confounding factors and reduce selection bias, we performed a propensity score matching between patients treated with BE and SE THVs according to the relevant anatomical features with an already known effect on TAVI outcome, such as aortic angulation and calcium volume 800 HU^5,14^.

To the best of our knowledge, this is the first attempt to elucidate the potential factors impacting on the final device implantation depth through a quantitative assessment of specific geometrical features of the device landing zone. Of note, the evaluation of both angulation and curvature of the aortic landing zone can be easily embedded in the planning of TAVI procedure; input data (aortic centerline and anatomical landmarks) can be directly extracted from software tools already available in clinical practice and the calculation process is not time-consuming.

Both angulation and curvature of the aortic landing zone led to a significant variation of implantation depth between the intended (i.e., measured at the non-recapture point for SE device) and the final one, upon complete release. Additionally, angulation of the distal landing zone arose as an independent predictor of increased implantation depth, also reporting at ROC analysis a cut off value equal to 17.8°.

**Figure 4:**
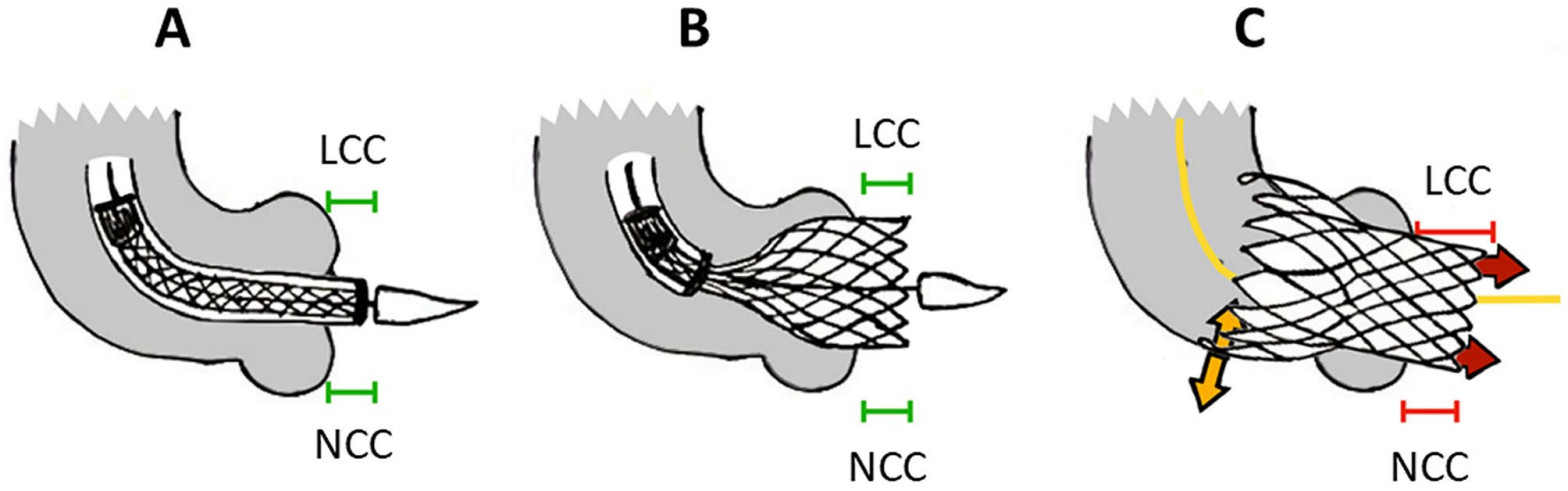
Mechanism of aortic landing zone interaction with SE valve. Initial positioning of the SE device with a symmetrical implantation depth on both NCC and LCC cusps (green bars, **A**); the intended implantation depth is maintained in the initial phase of SE release (**B**) while the final implantation depth of the device may change depending on the interaction of the device with the aortic wall anatomy (**C**). Abbreviations as in Fig. 2.

Furthermore, a different behavior was evident when comparing short-frame BE and high-frame SE THVs. On the one hand, BE devices systematically shortened during valve opening resulting in a final implantation depth slightly higher than the intended one, irrespectively of the degree of curvature and angulation of the landing zone. On the other hand, high-frame SE devices resulted in a final implantation depth deeper than the intended one, in particular at the LCC (Fig. 2). This may be due to the interaction of the upper part of the device with the proximal ascending aorta (Fig. 4), which locally exhibits higher values of curvature and angulation if compared to the AR region. Indeed, the initial, i.e., intended, positioning of the SE device (Fig. 4, panel A) is generally characterized on both NCC and LCC by a symmetrical implantation depth, which is preserved during the first phase of the device release (Fig. 4, panel B). However, in the final phase of the device release, the terminal portion of SE frame may directly interact with the aortic wall while realigning with the already deployed part of the device (Fig. 4, panel C). Accordingly, as also evident in Supplementary Video S1, a significant variation can be noticed for LCC implantation depth while this variation remains negligible for NCC side. Depending on the local angulation and curvature of the landing zone, mechanical interaction between the outer curve (i.e., NCC) of the ascending aorta and the prosthetic valve may occur, inducing a partial rotation of the device and increasing its axial motion along the contralateral (i.e., LCC) side of the landing zone.

**Figure 5:**
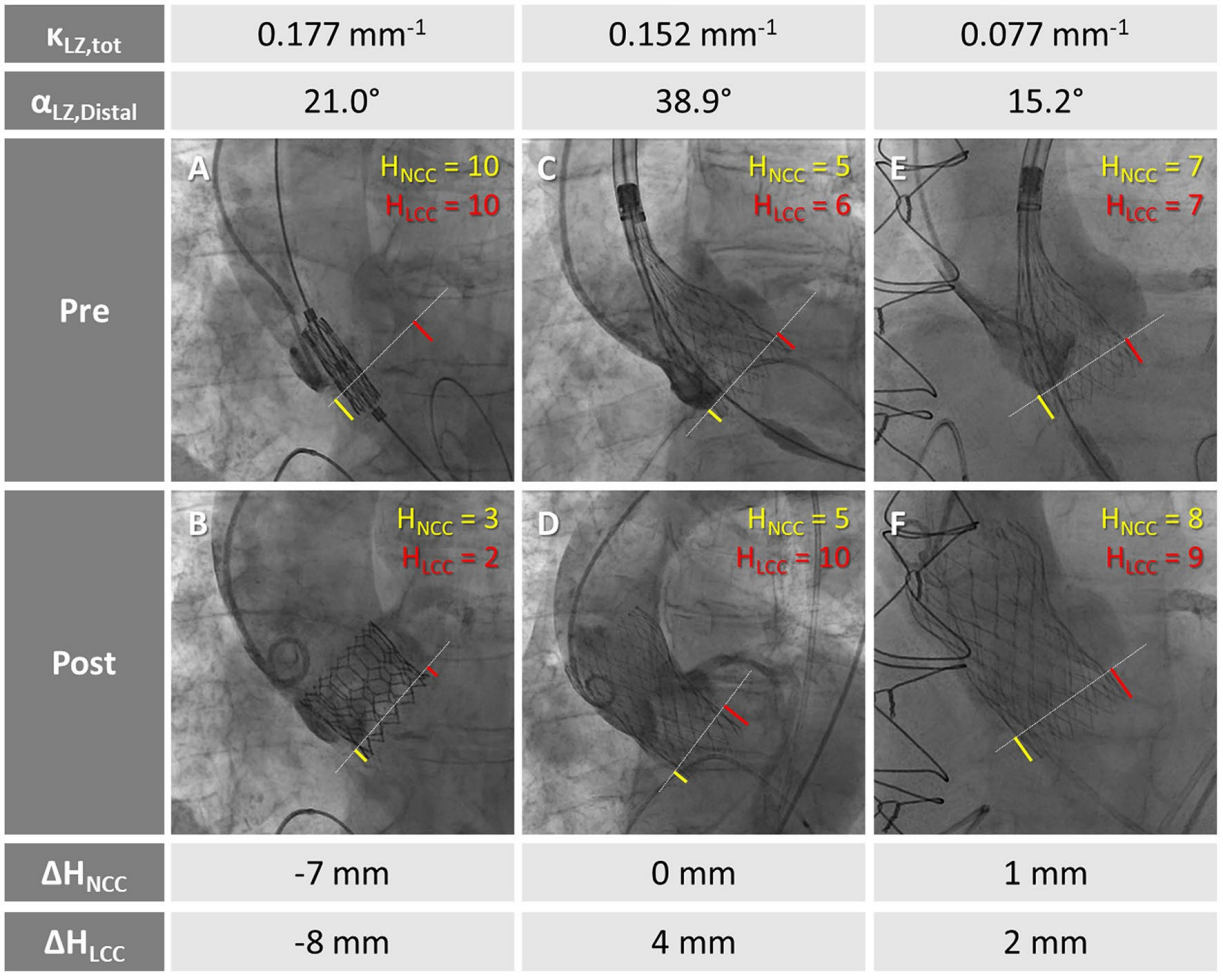
BE valve deployment in a patient with angulated proximal ascending aorta (**A**, **B**): (**A**) initial device positioning and (**B**) almost symmetrical higher final implantation depth (H) on both NCC and LCC cusps. SE valve implantation in a patient with high angulation of the proximal ascending aorta (**C**, **D**): (**C**) symmetrical initial implantation depth and (**D**) final tilted valve configuration with a deeper than expected implantation depth on LCC. SE valve implantation in a patient with restrained angulation of the proximal ascending aorta (**E**, **F**): (**E**) initial symmetrical THV positioning and (**F**) final THV implantation depth with symmetrical implantation depth on both LCC and NCC. Abbreviations as in Figs. 1 and 2.

For instance, in a patient with remarkable angulation and curvature of the proximal ascending aorta (Fig. 5, panel A), TAVI with BE valve generally reveals a symmetrical reduction of implantation depth on both LCC and NCC cusps since no mechanical interaction is expected between the device and the proximal ascending aorta (Fig. 5, panel B). Conversely, in patients referred to TAVI with SE devices, a pronounced angulation and curvature of the distal aortic landing zone can induce an axial motion and partial tilting of the device, in particular on LCC (Fig. 5, panels C and D) while this alteration in the final implantation depth is not evident with a less angulated distal aortic landing zone (Fig. 5, panels E and F).

The clinical implications of our findings may be relevant since the implantation depth is associated with the need of postprocedural PPI^15^ and PVL^5^, with both the conditions increasing the risk of all-cause mortality and cardiovascular mortality^7,16^.

Furthermore, there will be an increasing number of low-risk and younger patients undergoing TAVI^17,18^ in the future, in whom the need for postprocedural PPI will become less and less acceptable.

Thus, it will be of utmost importance to further improve procedural outcome. In this regard, the finding on CT scan of an angulated aortic landing zone may be relevant during preprocedural planning and should be interpreted as an anatomical feature supporting the choice of a short-frame device.

It is worth noting that Portico and Evolut-R THVs were grouped together within the high-frame SE group: despite a similar stent height, the Evolut-R stent frame is more rigid due the smaller cell design and has higher radial force as compared to the Portico^19^. Also, the EnVeo-R (Medtronic, Minneapolis, MN, USA) delivery system used for Evolut-R/Pro THV is made of a double spine technology, allowing steering only in two directions, while the FlexNav (Abbott, Minneapolis, MN, USA) delivery system used for Portico THV is made of a single spine technology and it is provided with a stabilization layer, which improves stability during valve release. Nonetheless, mismatch in mean implantation depth ${\Delta H}_{mean}$ proved to be comparable between Portico and Evolut sub-groups (*P* = 0.44, Figure S2), with statistically negligible differences on both LCC and NCC sides (*P* = 0.46 and *P* = 0.33, respectively).

There are main limitations that should be taken into consideration when interpreting the results of our analysis.

First, this is a proof-of-concept retrospective study investigating a potential relation between novel anatomical aortic features and implantation depth in TAVI based on a relatively small sample size, though we performed a propensity score match to account for possible bias in the selected population. Nonetheless, due to the available sample size, only 5 parameters (age, STS score, body surface area, calcium volume 800 HU and aortic angulation) were selected for propensity score matching; otherwise, the study population would have been too small. Nonetheless, several parameters not included in the one-to-one matching (e.g., prior atrial fibrillation, chronic obstructive pulmonary disease, coronary artery disease, prior coronary artery bypass grafting and prior acute myocardial infarction) were comparable between BE and SE datasets before matching and remained comparable also after one-to-one propensity matching.

Second, clinical validation of our findings with respect to current TAVI outcomes (i.e., PVL, PPI) should be investigated on a larger and prospective population study.

Third, calculation of the angulation and curvature of the aortic landing zone was performed through the combined use of different commercial tools. Nonetheless, the proposed metrics as well as the way they are calculated can be effectively automated and made accessible to clinicians in routine TAVI planning, directly embedding these measurements in commercial software already in use or further leveraging deep learning-based dedicated workflows^20^.

## Conclusions

During TAVI procedure, an increased angulation of the distal portion of THV landing zone proved to significantly impact on the final release of the device in terms of mismatch between the final and the intended implantation depth. Specifically, due to their remarkable frame height extending the surface of interaction with the proximal ascending aorta, the final implantation depth of SE devices may be deeper with respect to the intended one, in particular on LCC, as a consequence of the mechanical interplay between the device and the aortic wall in the final phase of device release.

### Supplementary Information

Supplementary Information 1.

Supplementary Video 1.

## Data Availability

The data that support the findings of this study will be available from the corresponding author upon reasonable request.

## Abbreviations

α_STJ_: Angulation of the STJ plane with respect to the aortic annulus plane
α_LZ__,Proximal_: Angulation of the proximal landing zone plane with respect to the aortic annulus plane
α_LZ__,Distal_: Angulation of the distal landing zone plane with respect to the aortic annulus plane
AA: Aortic angulation
AR: Aortic root
AUC: Area under the curve
BE: Balloon-expandable
BSA: Body surface area
CT: Computed tomography
H: Implantation depth
H_Pre_: Intended implantation depth
H_Post_: Final implantation depth
k_AR,tot_: Total curvature of the aortic root centerline
k_LZ,tot_: Total curvature of the landing zone centerline
L_AR_: Aortic root length
LCC: Left coronary cusp
LZ: Landing zone
MPR: Multiplanar reconstruction
NCC: Non-coronary cusp
PCI: Percutaneous coronary intervention
PPI: Permanent pacemaker implantation
PVL: Paravalvular leakage
ROC: Receiver-operating characteristic
SE: Self-expandable
STJ: Sinotubular junction
STS: Society of Thoracic Surgeons
TAVI: Transcatheter aortic valve implantation
THV: Transcatheter heart valve
∆H: Implantation depth mismatch
