# Supplementary material for: Angulation and curvature of aortic landing zone affect implantation depth in transcatheter aortic valve implantation
Source: Sci Rep. 2024 May 6;14:10409. doi: 10.1038/s41598-024-61084-5 (PMC11074135; doi:10.1038/s41598-024-61084-5)
Supplement: Supplementary file 1 — Supplementary Information 1. [file 41598_2024_61084_MOESM1_ESM.docx]

**Angulation and Curvature of Aortic Landing Zone Affect Implantation Depth in Transcatheter Aortic Valve Implantation**

Riccardo Gorla^1,^* MD, PhD, Omar A. Oliva^1^ MD, Luca Arzuffi^1^ MD, Valentina Milani^2^ PhD, Simone Saitta^3^ PhD, Mattia Squillace^1^ MD, Enrico Poletti^1^ MD, Maurizio Tusa^1^ MD, Emiliano Votta^3,4^, PhD Nedy Brambilla^1^ MD, Luca Testa^1^ MD, PhD, Francesco Bedogni^1^ MD^§^, Francesco Sturla^4,3^ PhD^§^

**^1^** Department of Clinical and Interventional Cardiology, IRCCS Policlinico San Donato, San Donato Milanese, Italy;

^2^ Scientific Directorate, IRCCS Policlinico San Donato, San Donato Milanese, Italy;

^3^ Department of Electronics, Information and Bioengineering, Politecnico di Milano, Milano, Italy;

^4^ 3D and Computer Simulation Laboratory, IRCCS Policlinico San Donato, San Donato Milanese, Italy;

^§^ Francesco Bedogni and Francesco Sturla are equally contributing principal investigators.

**Supplementary material**

***Corresponding author**

Riccardo Gorla, MD, PhD

Department of Clinical and Interventional Cardiology

IRCCS Policlinico San Donato, Milan, Italy

P.zza Edmondo Malan 2, 20097 San Donato Milanese, Milan, Italy

Mail: riccardo.gorla@grupposandonato.it

Phone: +39 02 5277 4980

**Supplementary Methods**

*Frenet-Serret formulas*

The Frenet-Serret theory of differential geometry describes the geometric properties of a 3D continuous curve, at least three times differentiable in the three-dimensional Euclidean space *R*^3^. The orthonormal basis $\left( \vec{T}(s),\vec{N}(s),\vec{B}(s) \right)$ is defined along the curve as:

| $\vec{T}(s)=\frac{\boldsymbol{r}^{'}\left( s \right)}{\left\Vert\boldsymbol{r}^{'}\left( s \right) \right\Vert}$ | (1) |
| --- | --- |
| $\vec{N}\left( s \right)=\frac{\vec{T}(s)}{\left\Vert\vec{T}(s) \right\Vert}=\frac{\boldsymbol{r}^{'}\left( s \right)\times\left( \boldsymbol{r}^{''}\left( s \right)\times\boldsymbol{r}^{'}\left( s \right) \right)}{\left\Vert\boldsymbol{r}^{'}\left( s \right) \right\Vert\left\Vert\boldsymbol{r}^{''}\left( s \right)\times\boldsymbol{r}^{'}\left( s \right) \right\Vert}$ | (2) |
| $\vec{B}\left( s \right)=\vec{T}\left( s \right)\times\vec{N}\left( s \right)=\frac{\boldsymbol{r}^{'}\left( s \right)\times\boldsymbol{r}^{''}\left( s \right)}{\left\Vert\boldsymbol{r}^{'}\left( s \right)\times\boldsymbol{r}^{''}\left( s \right) \right\Vert}$ | (3) |

where $\vec{T}$ is the unit vector tangent to the curve, $\vec{N}$ is the normal unit vector and $\vec{B}$ is the binormal unit vector; $\boldsymbol{r}^{'}(s)$, $\boldsymbol{r}^{''}(s)$ and $\boldsymbol{r}^{'''}(s)$ are the first, second and third derivates of the space curve. At each point of the curve, $\vec{T}(s)$ and $\vec{N}(s)$ vectors define the osculating plane.

At each point, curvature ($\kappa$) is defined as:

| $\kappa=\frac{\left\Vert\boldsymbol{r}^{'}\left( s \right)\times\boldsymbol{r}^{''}\left( s \right) \right\Vert}{\left\Vert\boldsymbol{r}^{'}\left( s \right) \right\Vert^{3}}$ | (4) |
| --- | --- |

where $\boldsymbol{r}^{'}(s)$ and $\boldsymbol{r}^{''}(s)$ are the first and second derivates of the curve and $\left\| \boldsymbol{r}^{'}\left( s \right) \right\|^{3}$ is the magnitude of their cross-product.

Torsion ($\tau$) is calculated from the Frenet-Serret differential formulae as:

| $\tau=\frac{\left[ \boldsymbol{r}^{'}\left( s \right),\boldsymbol{r}^{'}'\left( s \right),\boldsymbol{r}^{'''}\left( s \right) \right]}{\left\Vert\boldsymbol{r}^{'}\left( s \right)\times\boldsymbol{r}^{''}\left( s \right) \right\Vert^{2}}$ | (5) |
| --- | --- |

where $\left[ \boldsymbol{r}^{'}\left( s \right),\boldsymbol{r}^{'}'\left( s \right),\boldsymbol{r}^{'''}\left( s \right) \right]$ denotes a scalar triple product and $\boldsymbol{r}^{'''}(s)$ is the third derivate of the space curve.

Hence, curvature (*Equation 4*) represents the reciprocal of the radius of the osculating circle, which lies on the osculating plane with the center on $\vec{N}$; torsion (*Equation 5*) describes the rate of change in orientation of the osculating plane along each pathline.

**Supplementary Tables**

**Supplementary Table S1.** Nominal height of each TAVI device varied according to the valve size.

| **THV** | **Label size**  (mm) | **Frame height**  (mm) | **Inflow diameter**  (mm) | **Outflow diameter**  (mm) |
| --- | --- | --- | --- | --- |
| **Myval^§^** | 20 | 17.35 | - | - |
|  | 21.5 | 18.35 | - | - |
|  | 23 | 17.85 | - | - |
|  | 24.5 | 18.75 | - | - |
|  | 26 | 18.85 | - | - |
|  | 27.5 | 19.25 | - | - |
|  | 29 | 20.35 | - | - |
|  | 30.5 | 20.9 | - | - |
|  | 32 | 21.14 | - | - |
| **Sapien 3^§^** | 20 | 15.5 | - | - |
|  | 23 | 18 | - | - |
|  | 26 | 20 | - | - |
|  | 29 | 22.5 | - | - |
| **Portico** | 23 | 50 | 23 | 39 |
|  | 25 | 53 | 25 | 41 |
|  | 27 | 49 | 27 | 42 |
|  | 29 | 50 | 29 | 44 |
| **Evolut R/PRO** | 23 | 45 | 23 | 34 |
|  | 26 | 45 | 26 | 32 |
|  | 29 | 45 | 29 | 34 |
|  | 34 | 46 | 34 | 38 |
| **^§^**Inflow and outflow diameters equal to the specific THV label size. | | | | |

**Supplementary Table S2.** Summary of propensity score matching results.

| **Variables** | **Observations** | **Mean**  **Difference** | **Standard**  **deviation** | **Standardized**  **difference** | **Percent**  **Reduction** | **Variance Ratio** |
| --- | --- | --- | --- | --- | --- | --- |
| Score prop logit | All | 0.33 | 0.55 | 0.60 |  | 0.98 |
|  | Matched | -0.01 |  | -0.01 | 98.5 | 0.99 |
| Age (years) | All | -2.6 | 7.2 | -0.35 |  | 1.43 |
|  | Matched | -0.1 |  | -0.01 | 96.5 | 0.87 |
| BSA (m^2^) | All | 0.09 | 0.21 | 0.46 |  | 0.91 |
|  | Matched | -0.02 |  | -0.07 | 84.3 | 0.88 |
| Calcium volume 800 HU (mm^3^) | All | 73 | 355 | 0.21 |  | 1.12 |
|  | Matched | 30 |  | 0.08 | 59.0 | 0.88 |
| Angle (°) | All | -0.2 | 10.0 | -0.02 |  | 1.05 |
|  | Matched | 0.6 |  | 0.06 | 0.0 | 1.04 |
| STS score (%) | All | -0.5 | 3.4 | -0.16 |  | 1.04 |
|  | Matched | 0.2 |  | 0.06 | 62.9 | 0.99 |
| BSA, body surface area; HU, Hounsfield units; STS, Society of Thoracic Surgeons. | | | | | | |

**Supplementary Table S3.** Patient baseline main characteristics before (unmatched) and after (matched) propensity score matching.

| **Variables** | **Unmatched** | | | **matched** | | |
| --- | --- | --- | --- | --- | --- | --- |
|  | **BE**  (n = 143) | **SE**  (n = 641) | ***P* value** | **BE**  (n = 133) | **SE**  (n = 133) | ***P* value** |
| Age (years) | 81 (77, 85) | 84 (79, 87) | **< 0.001** | 81 (78, 86) | 82 (77, 86) | 0.93 |
| Female sex | 40 (28.0) | 366 (57.1) | **< 0.001** | 37 (27.8) | 52 (39.1) | 0.07 |
| BSA (m^2^) | 1.85 ± 0.20 | 1.75 ± 0.21 | **< 0.001** | 1.84 ± 0.20 | 1.86 ± 0.21 | 0.64 |
| Hypertension | 107 (74.8) | 490 (76.4) | 0.67 | 101 (75.9) | 96 (72.2) | 0.58 |
| Diabetes | 44 (30.8) | 178 (27.8) | 0.47 | 41 (30.8) | 37 (27.8) | 0.69 |
| Dyslipidemia | 54 (37.8) | 258 (40.3) | 0.64 | 58 (43.6) | 51 (38.4) | 0.45 |
| COPD | 17 (11.9) | 96 (15.0) | 0.43 | 16 (12.0) | 21 (15.8) | 0.48 |
| Coronary artery disease | 29 (20.3) | 150 (23.4) | 0.44 | 27 (20.3) | 37 (27.8) | 0.20 |
| Prior AF | 44 (30.8) | 164 (25.6) | 0.21 | 41 (38.8) | 36 (27.1) | 0.59 |
| Prior CABG | 12 (8.4) | 73 (11.4) | 0.37 | 11 (8.3) | 20 (15.0) | 0.13 |
| Prior AMI | 12 (8.4) | 55 (8.6) | 0.99 | 12 (9.0) | 9 (6.8) | 0.65 |
| STS score (%) | 3.1 (2.2, 5.7) | 4.1 (2.7, 6.3) | **0.001** | 3.1 (2.3, 5.7) | 3.2 (2.0, 5.3) | 0.60 |
| Creatinine clearance (mL/min/1.73 m^2^) | 63.0 (48.5, 81.0) | 56.0 (42.0, 72.0) | **0.002** | 62.0 (47.0, 80.5) | 57.0 (43.0, 75.0) | 0.08 |
| Haemoglobin (g/dL) | 12.5 ± 2.0 | 12.3 ± 1.9 | 0.20 | 12.5 ± 2.0 | 12.4 ± 1.8 | 0.56 |
| Ejection fraction (%) | 52.0 (41.5, 60.0) | 57.0 (49.0, 64.0) | **< 0.001** | 52.0 (42.5, 60.0) | 59.0 (50.0, 65.0) | **< 0.001** |
| Mean AV gradient (mmHg) | 41.0 (32.0, 49.0) | 43.0 (35.0, 52.0) | **0.02** | 40.5 (31.3, 49.0) | 45.0 (36.0, 56.0) | **0.005** |
| Aortic regurgitation ≥ moderate | 28 (19.7) | 102 (15.9) | 0.35 | 22 (16.8) | 19 (14.4) | 0.72 |
| Calcium volume 800 HU (mm^3^) | 268 (132, 482) | 223 (113, 418) | **0.04** | 265 (126, 512) | 197 (107, 454) | 0.20 |
| Aortic angulation (°) | 48.7 ± 10.0 | 48.5 ± 9.9 | 0.86 | 48.7 ± 10.2 | 48.1 ± 10.0 | 0.63 |
| Values are mean ± SD, median (IQR) or n (% of column total)  AF, atrial fibrillation; AMI, acute myocardial infarction; BE, balloon-expandable; BSA, body surface area; CABG, coronary artery bypass grafting; COPD, chronic obstructive pulmonary disease; HU, Hounsfield Units; SE, self-expandable; STS, Society of Thoracic Surgeons. | | | | | | |

**Supplementary Table S4.** Baseline patient characteristics based on the mean variation of the implantation height (${\Delta H}_{mean}$).

| **Variables** | $\boldsymbol{\Delta H}_{\boldsymbol{mean}}$ | | ***P* Value** |
| --- | --- | --- | --- |
|  | $\boldsymbol{<}{\bar{\boldsymbol{\Delta H}}}_{\boldsymbol{mean}}$  (n = 94) | $\boldsymbol{\geq}{\bar{\boldsymbol{\Delta H}}}_{\boldsymbol{mean}}$  (n = 108) |  |
| BE | 85 (90.4) | 16 (14.8) | **<0.001** |
| SE | 9 (9.6) | 92 (85.2) | **<0.001** |
| Age (years) | 81 (77, 85) | 82 (78, 86) | 0.97 |
| Female sex | 26 (27.7) | 38 (35.2) | 0.25 |
| Hypertension | 69 (73.4) | 82 (75.9) | 0.68 |
| Diabetes | 24 (25.5) | 33 (30.6) | 0.43 |
| Dyslipidemia | 38 (40.4) | 43 (39.8) | 0.93 |
| COPD | 13 (13.8) | 14 (13.0) | 0.86 |
| CAD | 21 (22.3) | 27 (25.0) | 0.74 |
| Prior CABG | 9 (9.6) | 14 (13.0) | 0.51 |
| Prior AMI | 8 (8.5) | 7 (6.5) | 0.58 |
| Prior AF | 26 (27.7) | 32 (29.6) | 0.31 |
| STS score (%) | 3.2 (2.4, 5.6) | 3.2 (2.1, 5.6) | 0.63 |
| Creatinine clearance (mL/min/1.73 m^2^) | 62.0  (46.0, 81.0) | 56.0  (42.0, 71.0) | 0.06 |
| Haemoglobin (g/dL) | 12.8 ± 1.9 | 12.3 ± 1.9 | 0.08 |
| Ejection fraction (%) | 51.0 (40.5, 60.0) | 58.0 (50.0, 65.0) | **0.005** |
| Mean AV gradient (mmHg) | 39.2 ± 15.0 | 45.4 ± 14.5 | **0.005** |
| AR ≥ moderate | 20 (21.3) | 15 (13.9) | 0.19 |
| LM height (mm) | 16.0 ± 4.2 | 15.6 ± 3.4 | 0.41 |
| RCA height (mm) | 19.7 ± 3.4 | 19.2 ± 3.8 | 0.31 |
| Annulus minimum diameter (mm) | 23.1 ± 2.8 | 21.8 ± 3.0 | **0.002** |
| Annulus maximum diameter (mm) | 29.1 ± 3.0 | 27.8 ± 2.6 | **<0.001** |
| Annulus mean diameter (mm) | 26.1 ± 2.8 | 24.8 ± 2.4 | **<0.001** |
| Annulus perimeter (mm) | 82.0 ± 8.7 | 78.2 ± 7.3 | **<0.001** |
| Annulus area (mm^2^) | 523.4 ± 111.6 | 473.0 ± 89.6 | **<0.001** |
| LVOT diameter (mm) | 25.8 ± 3.3 | 24.2 ± 3.0 | **<0.001** |
| Valsalva diameter (mm) | 34.6 ± 3.8 | 33.5 ± 3.7 | 0.052 |
| Calcium volume 800 HU  (mm^3^) | 274 (120, 530) | 211 (116, 433) | 0.11 |
| Aortic angulation (°) | 48.0 ± 9.9 | 48.2 ± 9.2 | 0.89 |
| Index of eccentricity | 0.20 (0.17, 0.24) | 0.21 (0.17, 0.26) | 0.56 |
| L_AR_ (mm) | 23.2 ± 3.7 | 21.7 ± 3.4 | **0.003** |
| k_AR,tot_ (10^-1^·mm^-1^) | 0.41 (0.27, 0.53) | 0.35 (0.26, 0.46) | 0.07 |
| k_LZ,tot_ (10^-1^·mm^-1^) | 0.47 (0.27, 0.65) | 1.18 (0.88, 1.46) | **<0.001** |
| α_STJ_ (°) | 10.5 (6.2, 13.9) | 8.5 (5.1, 12.6) | 0.06 |
| α_LZ,proximal_ (°) | 2.8 (1.6, 4.5) | 2.5 (1.3, 4.1) | 0.31 |
| α_LZ,distal_ (°) | 7.8 (5.0, 12.7) | 27.2 (18.9, 33.9) | **<0.001** |
| Values are mean ± SD, median (IQR) or n (% of column total). ^§^Group $<\bar{\Delta H}_{mean}$ vs. group $\boldsymbol{\geq}\bar{\Delta H}_{mean}$.  AMI, acute myocardial infarction; AR, aortic root; AV, aortic valve; BE, balloon-expandable; CABG, coronary artery bypass grafting; COPD, chronic obstructive pulmonary disease; HU, Hounsfield Units; k_AR,tot_, total (cumulative) curvature of the aortic root centerline; k_LZ,tot_, total (cumulative) curvature of the landing zone centerline; LZ, landing zone; L_AR_, aortic root length; LM, left main; LVOT, left ventricular outflow tract; RCA, right coronary artery; SE, self-expandable; STJ, sinotubular junction; STS, Society of Thoracic Surgeons; α_STJ_, angulation of the STJ plane with respect to the aortic annulus plane; α_LZ,Proximal_, angulation of the proximal LZ plane with respect to the aortic annulus plane; α_LZ,Distal_, angulation of the distal LZ plane with respect to the aortic annulus plane | | | |

**Supplementary Table S5.** Procedural data and clinical in-hospital outcome based on the mean variation of the implantation height (${\Delta H}_{mean}$).

| **Variables** | $\boldsymbol{\Delta H}_{\boldsymbol{mean}}$ | | ***P* Value** |
| --- | --- | --- | --- |
|  | $\boldsymbol{<}{\bar{\boldsymbol{\Delta H}}}_{\boldsymbol{mean}}$  (n = 94) | $\boldsymbol{\geq}{\bar{\boldsymbol{\Delta H}}}_{\boldsymbol{mean}}$  (n=108) |  |
| Implanted valve type |  |  |  |
| Myval | 70 (74.5) | 11 (10.2) | **<0.001** |
| Sapien 3 | 15 (16.0) | 5 (4.6) | **<0.001** |
| Evolut Pro | 4 (4.3) | 18 (16.7) | **<0.001** |
| Evolut R | 2 (2.1) | 51 (47.2) | **<0.001** |
| Portico | 3 (3.2) | 23 (21.3) | **<0.001** |
| Femoral route | 83 (88.3) | 96 (88.9) | 0.99 |
| Subclavian route | 3 (3.2) | 8 (7.4) | 0.23 |
| EPS | 2 (2.1) | 3 (2.8) | 0.99 |
| Any vascular complications | 6 (6.4) | 5 (4.6) | 0.58 |
| PTA with stenting of access site | 6 (6.4) | 4 (3.7) | 0.52 |
| PCI with stenting | 11 (11.7) | 6 (5.6) | 0.13 |
| Predilatation | 34 (36.2) | 48 (44.4) | 0.25 |
| Implantation depth |  |  |  |
| NCC H_Pre_ (mm) | 8.0 (7.0, 10.0) | 6.0 (4.3, 7.0) | **<0.001** |
| LCC H_Pre_ (mm) | 9.0 (8.0, 11.0) | 7.0 (5.0, 8.0) | **<0.001** |
| NCC H_Post_ (mm) | 4.0 (3.0, 6.0) | 7.0 (5.0, 9.0) | **<0.001** |
| LCC H_Post_ (mm) | 4.0 (3.0, 5.0) | 8.0 (6.0, 10.0) | **<0.001** |
| NCC ∆H (mm) | -4.0 (-6.0, -3.0) | 0.0 (-1.0, 2.0) | **<0.001** |
| LCC ∆H (mm) | -5.0 (-7.0, -3.0) | 1.0 (-0.8, 3.0) | **<0.001** |
| ${\Delta H}_{mean}$ (mm) | -4.7 ± 2.0 | 1.2 ± 2.3 | **<0.001** |
| Postdilatation | 8 (8.5) | 50 (46.3) | **<0.001** |
| Emergent cardiac surgery | 0 (0.0) | 0 (0.0) | - |
| Need for second valve | 0 (0.0) | 0 (0.0) | - |
| Contrast volume (mL) | 150 (115, 180) | 150 (120, 180) | 0.48 |
| Radiation time (min) | 18.8 (14.1, 23.6) | 21.3 (15.9, 27.8) | **0.04** |
| In-hospital outcome |  |  |  |
| Ejection fraction (%) | 55.0 (45.0, 63.0) | 58.0 (51.0, 64.0) | 0.12 |
| Mean gradient (mmHg) | 7.0 (6.0, 9.0) | 7.0 (5.0, 10.0) | 0.39 |
| PVL absent/trivial | 55 (58.5) | 34 (31.5) | **<0.001** |
| PVL mild | 31 (33.0) | 58 (53.7) | **0.004** |
| PVL > moderate | 4 (4.3) | 13 (12.0) | 0.07 |
| Device success | 86 (91.5) | 101 (93.5) | 0.60 |
| PPI | 13 (13.8) | 18 (16.7) | 0.70 |
| Stroke* | 1 (1.1) | 3 (2.8) | 0.62 |
| In-hospital mortality | 1 (1.1) | 0 (0.0) | 0.47 |
| Values are mean ± SD, median (IQR) or n (% of column total). Variation of implantation depth (∆H) calculated as H_Post_ - H_Pre_; * Stroke including not disabling.  ^§^Group $<\bar{\Delta H}_{mean}$ vs. group $\boldsymbol{\geq}\bar{\Delta H}_{mean}$.  EPS, embolic protection system; H_Pre_, final implantation depth; H_Pre_, pre-implantation intended depth; LCC, left coronary cusp; NCC, non-coronary cusp; PCI, percutaneous coronary intervention; PPI, permanent pacemaker implantation; PTA, percutaneous transluminal angioplasty; PVL, paravalvular leakage; ∆H, mismatch in implantation depth. | | | |

**Supplementary Table S6.** Receiver-operating characteristic (ROC) classification table for the standard logistic regression performed for each aortic feature and LZ parameter.

| **Variable** | **Actual** $\boldsymbol{\Delta H}_{\boldsymbol{mean}}$ | **Predicted** $\boldsymbol{\Delta H}_{\boldsymbol{mean}}$ | | **Sensitivity** | **Specificity** | **Accuracy** |
| --- | --- | --- | --- | --- | --- | --- |
|  |  | $\boldsymbol{\geq}{\bar{\boldsymbol{\Delta H}}}_{\boldsymbol{mean}}$ | ${\boldsymbol{<}\bar{\boldsymbol{\Delta H}}}_{\boldsymbol{mean}}$ |  |  |  |
| Calcium score 800 HU | $\geq\bar{\Delta H}_{mean}$ | 92 | 16 | 85.2 | 22.3 | 55.9 |
|  | ${<\bar{\Delta H}}_{mean}$ | 73 | 21 |  |  |  |
| Aortic angulation | $\geq\bar{\Delta H}_{mean}$ | 108 | 0 | 100 | 0.0 | 53.5 |
|  | ${<\bar{\Delta H}}_{mean}$ | 94 | 0 |  |  |  |
| Annulus minimal diameter | $\geq\bar{\Delta H}_{mean}$ | 74 | 34 | 68.5 | 48.9 | 59.4 |
|  | ${<\bar{\Delta H}}_{mean}$ | 48 | 46 |  |  |  |
| Annulus mean diameter | $\geq\bar{\Delta H}_{mean}$ | 76 | 32 | 70.4 | 57.4 | 64.4 |
|  | ${<\bar{\Delta H}}_{mean}$ | 40 | 54 |  |  |  |
| Annulus maximal diameter | $\geq\bar{\Delta H}_{mean}$ | 77 | 31 | 71.3 | 53.2 | 62.9 |
|  | ${<\bar{\Delta H}}_{mean}$ | 44 | 50 |  |  |  |
| Annulus perimeter | $\geq\bar{\Delta H}_{mean}$ | 77 | 31 | 71.3 | 56.4 | 64.4 |
|  | ${<\bar{\Delta H}}_{mean}$ | 41 | 53 |  |  |  |
| Annulus area | $\geq\bar{\Delta H}_{mean}$ | 76 | 32 | 70.4 | 55.3 | 63.4 |
|  | ${<\bar{\Delta H}}_{mean}$ | 42 | 52 |  |  |  |
| LVOT diameter | $\geq\bar{\Delta H}_{mean}$ | 68 | 36 | 65.4 | 57.4 | 61.6 |
|  | ${<\bar{\Delta H}}_{mean}$ | 40 | 54 |  |  |  |
| Valsalva diameter | $\geq\bar{\Delta H}_{mean}$ | 79 | 26 | 75.2 | 34.0 | 55.8 |
|  | ${<\bar{\Delta H}}_{mean}$ | 62 | 32 |  |  |  |
| L_AR_ | $\geq\bar{\Delta H}_{mean}$ | 76 | 32 | 70.4 | 41.5 | 56.9 |
|  | ${<\bar{\Delta H}}_{mean}$ | 55 | 39 |  |  |  |
| k_AR,tot_ | $\geq\bar{\Delta H}_{mean}$ | 93 | 15 | 86.1 | 22.3 | 56.4 |
|  | ${<\bar{\Delta H}}_{mean}$ | 73 | 21 |  |  |  |
| k_LZ,tot_ | $\geq\bar{\Delta H}_{mean}$ | 88 | 20 | 81.5 | 81.9 | 81.7 |
|  | ${<\bar{\Delta H}}_{mean}$ | 17 | 77 |  |  |  |
| α_STJ_ | $\geq\bar{\Delta H}_{mean}$ | 88 | 20 | 81.5 | 25.5 | 55.4 |
|  | ${<\bar{\Delta H}}_{mean}$ | 70 | 24 |  |  |  |
| α_LZ,Proximal_ | $\geq\bar{\Delta H}_{mean}$ | 94 | 14 | 87.0 | 16.0 | 54.0 |
|  | ${<\bar{\Delta H}}_{mean}$ | 79 | 15 |  |  |  |
| α_LZ,Distal_ | $\geq\bar{\Delta H}_{mean}$ | 88 | 20 | 81.5 | 86.2 | 83.7 |
|  | ${<\bar{\Delta H}}_{mean}$ | 13 | 81 |  |  |  |
| True positive (TP): actual and predicted ${\Delta H}_{mean}$ $\geq\bar{\Delta H}_{mean}$  True negative (TN): actual and predicted ${\Delta H}_{mean}$ $<\bar{\Delta H}_{mean}$  False positive (FP): predicted ${\Delta H}_{mean}$ $\geq\bar{\Delta H}_{mean}$ but actual ${\Delta H}_{mean}$ $<\bar{\Delta H}_{mean}$  False negative (FN): predicted ${\Delta H}_{mean}$ $<\bar{\Delta H}_{mean}$ but actual ${\Delta H}_{mean}$ $\geq\bar{\Delta H}_{mean}$  Sensitivity = TP/(TP+FN); specificity = TN/(TN+FP); accuracy = (TP+TN)/(TP+FN+TN+FP). | | | | | | |

**Supplementary Figures**

**Supplementary Figure S1**

Flow chart of study population and final analysis cohort.

**
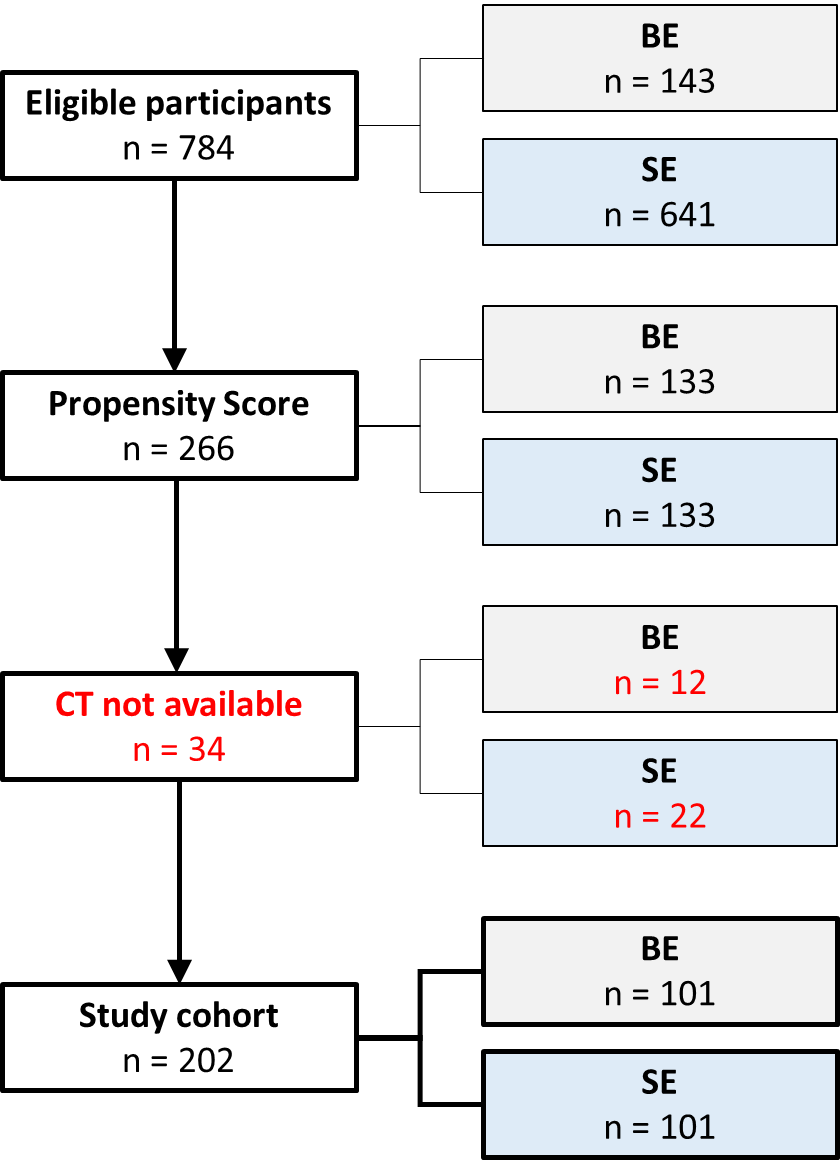
**

**Supplementary Figure S2**

Mismatch in the mean implantation depth ${\Delta H}_{mean}$ for Portico and Evolut SE devices reported as mean, NCC and LCC values.


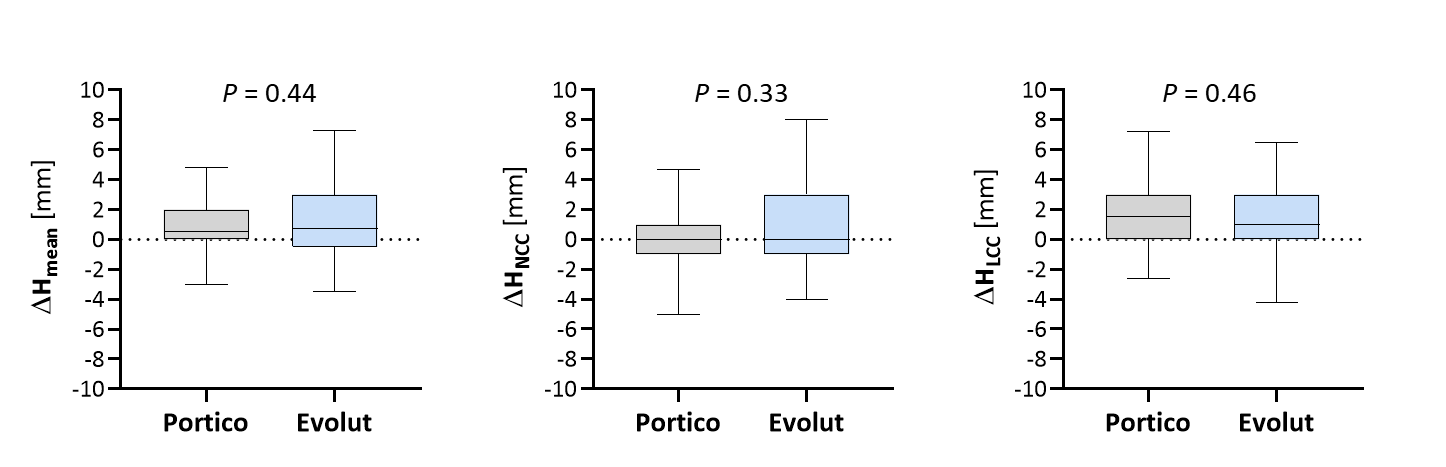


**Supplementary Video S1**

High-frame SE valve implantation: a symmetrical implantation depth is visible when the valve is released up to the no recapture point; after complete valve release, as well as at final angiography, the fully deployed valve configuration exhibits a deeper than expected implantation depth on LCC.
